# Editorial: Genomic Selection: Lessons Learned and Perspectives

**DOI:** 10.3389/fpls.2022.890434

**Published:** 2022-05-27

**Authors:** Johannes W. R. Martini, Sarah J. Hearne, Brian Gardunia, Valentin Wimmer, Fernando H. Toledo

**Affiliations:** ^1^International Maize and Wheat Improvement Center, Texcoco, Mexico; ^2^Excellence in Breeding Platform, Texcoco, Mexico; ^3^Bayer Crop Science, St. Louis, MO, United States; ^4^KWS SAAT SE & Co., KGaA, Einbeck, Germany

**Keywords:** genomic selection (GS), plant breeding, selection gain, breeding schemes, genotype-by-environment interaction

Genomic selection (GS) has been one of the most prominent Research Topics in breeding science in the last two decades after the milestone paper by Meuwissen et al. (2001). Its huge potential for increasing the efficiency of breeding programs attracted scientific curiosity and research funding. Many different statistical prediction methods have been tested, and different use cases have been explored.

We organized this Research Topic to look both back and forward. The objectives were to review the developments of the last 20 years, to provide a snapshot of current hot topics, and potentially also to define areas on which more (or less) focus should be put in the future, thereby supporting readers with formulating and prioritizing their ideas for future research.

Several questions were brought up when organizing this Research Topic including: How did GS change breeding schemes? Which impact did GS have on realized selection gain? What, considering the context of particularities of different crops, may be optimal breeding schemes to leverage the full potential of GS? What has been the impact of and what is the potential of hybrid prediction, statistical epistasis models, deep learning and other methods? What are the long-term effects of GS? Can predictive breeding approaches also be used to harness genetic resources from germplasm banks in a more efficient way?

Having closed our Research Topic, we are happy to present a solid collection of 21 contributions from 149 authors which reviews the past work around GS, presents new insights, and points at topics with potential for future research. The 21 contributions consist of 12 original research articles, a method paper, two review contributions, five opinion articles and a perspective.

Concerning original research, the main topics that have been addressed were “genetic architecture” and “genetic architecture enhanced prediction methods,” “shortening the breeding cycle,” “genotype x environment interaction,” “sparse-testing,” and “genomic selection in polyploids.”

Additionally to considerations around GS for major staple crops, Ferrão et al. “propose a strategy for using genomic selection in blueberry, with the potential to be applied to other polyploid species of a similar background.” In particular, the authors highlight that “the use of additive effects under a linear mixed model framework (GBLUP) showed the best balance between efficiency and accuracy.” The topic of GS in tetraploids has also been considered by Wilson et al. for the case of potato. Moreover, Liu et al. investigated prediction methods based on genes known to be relevant for fiber length in cotton. Pégard et al. considered GS for poplar in the context of forest tree breeding and highlight “that genomic evaluation performance could be comparable to the already well-optimized pedigree-based evaluation under certain conditions […] Genome-based methods showed advantages over pedigree counterparts when ranking candidates at the within-family levels, for most of the families.”

The other eight original research contributions were related to wheat, maize and rice.

Bonnett et al. addressed the application of GS in a wheat breeding pipeline. In particular, the authors considered the performance of selected material when applying genomic selection with different prediction methods in an early generation.

The topic of modeling environmental effects and genotype-by-environment interactions (GEI) was addressed by several authors. Westhues et al. included environmental predictors in GS using gradient boosting. Based on “data collected by the Maize Genomes to Fields” initiative, the authors found that “Accuracy in forecasting grain yield performance of new genotypes in a new year was improved by up to 20% over the baseline model by including environmental predictors with gradient boosting methods.” Genotype-by-environment interactions were also considered by Tomar et al. who investigated the predictive ability of a multi-environment genomic prediction model for yield in spring wheat. Atanda et al. and He et al. considered the modeling of GEI with the focus on applications in sparse-testing, and Rembe et al. investigated the impact of GEI on reciprocal recurrent genomic selection.

Ma and Cao addressed the dissection of grain yield of maize and compared the predictive ability of different approaches, in particular when incorporating markers associated with the traits of interest as a fixed effect in the statistical model. Finally, Cao et al. addressed genomic prediction of resistance to Tar Spot.

As a contribution of a method article, Schrauf et al. discussed how to compare different genomic prediction models by cross validations. The authors “emphasize the importance of paired comparisons to achieve high power in the comparison between candidate models, as well as the need to define notions of relevance in the difference between their performances. Regarding the latter,” the authors “borrow the idea of equivalence margins from clinical research and introduce new statistical tests.”

As review contributions, Fritsche-Neto et al. reviewed GS in small scale maize hybrid programs and Simeão et al. described the current status and future application of GS in tropical forage grasses.

Concerning opinion articles, Crossa et al. presented their view on the “Modern Plant Breeding Triangle,” comprising genomics, phenomics, and environomics. Martini et al. highlighted the challenges that prediction approaches face when aiming at harnessing genetic resources, that is predicting diverse material which may not be sufficiently represented in the training set. Covarrubias-Pazaran et al. outlined how public breeding programs could be strengthened by focusing on quantitative genetics principles, and by sharing data resources including genomic data and breeding values predicted from experimental evaluations from different organizations. Another opinion contribution was provided by Gholami et al. who compared the adoption of GS across different breeding institutions, in more detail dairy cattle breeding and public and private plant breeding programs. The authors highlight that differences in the organizational structure of plant and animal breeding institutions, as well as differences in the cost-benefit structures of the use of GS in private and public plant breeding may have been the cause for differences in the adoption of GS. Gianola contributed with his reflections on trends and developments in statistical genetics addressing for instance the “deconstruction of genetic architecture” and highlighting that “quantitative genetics provides just a linear (local) approximation to complexity with little (if any) mechanistic value.” Moreover, the author emphasized the principal of parsimony in genetic models and that a bias of a statistical method does not need to be a problem but that “practically all machine learning methods (e.g., random forests) provide biased predictions that, on average, will be better than unbiased machines.”

In the direction of what Gianola called the “linear (local) approximation,” Powell et al. argue that “The implicit capture of non-stationary effects of alleles requires the G2P map to be re-estimated across different contexts” and discuss the “development and application of hierarchical G2P maps that explicitly capture non-stationary effects of alleles.”

The rough outline of the content of our Research Topic emphasizes that GS is now well-established across many plant species. Moreover, five out of 12 research articles were related to GEI indicating the relevance of this topic in current research. Plant breeding programs may have more need to estimate GEI because a program's purpose is to develop improved varieties which is inherently tied to the target environments. Was our Research Topic able to answer all the questions originally formulated? We do not think so. For instance, additional contributions on the optimal use of GS for different crops, but also a more detailed retrospective analysis of realized selection gain after the introduction of GS, or the relevance of epistasis models, hybrid prediction and new machine learning models would have been desirable.

We hope that our Research Topic supports readers with the priorization of their own ideas for future investigation, and we look forward to a potential second volume, maybe 25 years after the milestone paper by Meuwissen et al. (2001).

## Author Contributions

JM drafted the Editorial. SH, BG, VW, and FT checked the manuscript and suggested modifications. All authors contributed to the article and approved the submitted version.

## Conflict of Interest

VW was employed by KWS SAAT SE & Co., KGaA, Einbeck, Germany. BG was employed by Bayer Crop Science, St. Louis, MO, United States. The remaining authors declare that the research was conducted in the absence of any commercial or financial relationships that could be construed as a potential conflict of interest.

## Publisher's Note

All claims expressed in this article are solely those of the authors and do not necessarily represent those of their affiliated organizations, or those of the publisher, the editors and the reviewers. Any product that may be evaluated in this article, or claim that may be made by its manufacturer, is not guaranteed or endorsed by the publisher.
